# Mitochondrial DNA Rearrangement Spectrum in Brain Tissue of Alzheimer’s Disease: Analysis of 13 Cases

**DOI:** 10.1371/journal.pone.0154582

**Published:** 2016-06-14

**Authors:** Yucai Chen, Changsheng Liu, William Davis Parker, Hongyi Chen, Thomas G. Beach, Xinhua Liu, Geidy E. Serrano, Yanfen Lu, Jianjun Huang, Kunfang Yang, Chunmei Wang

**Affiliations:** 1 Neurology Department, Shanghai Children’s Hospital, Shanghai Jiao Tong University, Shanghai, China; 2 Pediatric Department, University of Illinois at Chicago, Peoria, United States of America; 3 SoftGenetics LLC, State College, United States of America; 4 Banner Sun Health Research Institute, Sun City, United States of America; Ben-Gurion University of the Negev, ISRAEL

## Abstract

**Background:**

Mitochondrial dysfunction may play a central role in the pathologic process of Alzheimer’s disease (AD), but there is still a scarcity of data that directly links the pathology of AD with the alteration of mitochondrial DNA. This study aimed to provide a comprehensive assessment of mtDNA rearrangement events in AD brain tissue.

**Patients and Methods:**

Postmortem frozen human brain cerebral cortex samples were obtained from the Banner Sun Health Research Institute Brain and Body Donation Program, Sun City, AZ. Mitochondria were isolated and direct sequence by using MiSeq®, and analyzed by relative software.

**Results:**

Three types of mitochondrial DNA (mtDNA) rearrangements have been seen in post mortem human brain tissue from patients with AD and age matched control. These observed rearrangements include a deletion, F-type rearrangement, and R-type rearrangement. We detected a high level of mtDNA rearrangement in brain tissue from cognitively normal subjects, as well as the patients with Alzheimer's disease (AD). The rate of rearrangements was calculated by dividing the number of positive rearrangements by the coverage depth. The rearrangement rate was significantly higher in AD brain tissue than in control brain tissue (17.9%versus 6.7%; p = 0.0052). Of specific types of rearrangement, deletions were markedly increased in AD (9.2% versus 2.3%; p = 0.0005).

**Conclusions:**

Our data showed that failure of mitochondrial DNA in AD brain might be important etiology of AD pathology.

## Introduction

Research suggests that mitochondrial changes are a driving force, rather than a consequence, of the aging process and AD pathogenesis [1]. Although point mutations of mitochondrial DNA have been hypothesized as being a critical cause of aging, there is evidence that they may not be fully explanatory [2]. Mitochondria are dynamic organelles with very short half-lives. Continuous replication of mitochondrial DNA is required for assignment to new mitochondria, resulting in a significant error rate and accumulation of mutated in mtDNA genome over time and space. We hypothesized that, beyond point mutations, different types of mtDNA rearrangements should be extensively distributed in aging cells. As these rearrangements are often not detected by routine methods such as polymerase chain reaction, we applied approach by directly sequencing mtDNA from isolated mitochondria of fresh frozen brain samples and count. Our data show that different types of mitochondrial rearrangements are very common in both aging brain and Alzheimer’s disease (AD) brain. We classified observed mitochondrial DNA rearrangements into three types: deletion, F-type rearrangement and R-type rearrangement. We also found that brain tissue of subjects with AD had 2.7 times the rearrangement rate of brain tissue from age-similar non-demented (ND) control subjects.

## Materials and Methods

### Human subjects

Brain tissues were obtained from the Banner Sun Health Research Institute Brain and Body Donation Program (BBDP), Sun City, AZ [3,4]. The operations of the Brain and Body Donation Program are approved by the designated Banner Sun Health Research Institute Institutional Review Board. All subjects or their legal representatives signed the informed consent. The experimental methods were carried out in accordance with the approved guidelines. Human subjects had been diagnosed with dementia during life and met National Institute on Aging Reagan Institute intermediate or high criteria for AD after autopsy and neuropathological examination [5]. Control subjects were non-demented during life and had low densities of AD histopathology. Control and AD subjects did not significantly differ (Table 1) in terms of postmortem interval (PMI) or age at death. As expected by diagnosis, control subjects had significantly higher Mini Mental State Examination (MMSE) scores and significantly lower densities of neuritic plaques [6,7].

**Table 1:** Characteristics of human subjects.

Diagnosis (N)	Age (SD)	PMI	MMSE	NP density	Braak
Control (12)	81.9 (10.3)	3.0 (0.74)	28.0 (1.8)^1^	0.8 (0.4)	1.8 (1.0)
AD (13)	81.1 (9.2)	2.8 (0.6)	9.5 (10.1)^2^	3.0 (0.0)	5.4 (0.5)

Note: Means and standard deviations (SD) are given. PMI = postmortem interval; MMSE = last Mini Mental State Examination Score; NP density = CERAD neuritic plaque density; Braak = Braak neurofibrillary tangle stage.

### Mitochondrial DNA isolation

Mitochondria were isolated from approximately 250 mg of gray matter from the frontal lobe using a Mitochondria Isolation Kit for Tissue (Thermo Scientific), followed suggested protocol by company using option B. Afterwards, DNA was extracted using a QIAprep Spin Miniprep Kit (Qiagen). After DNA extraction, any co-extracted mitochondrial RNA was removed by the addition of RNase A. Using this procedure, we can extract up to 10ng mtDNA from 250mg of brain tissue, which is enough for direct sequencing (1ng required for Nextera XT kit, Illumina). We used the Nextera XT DNA Sample Prep Kit (Illumina)to prepare DNA samples for sequencing followed by the MiSeq® Reagent Kit v3 (600 cycle), run on paired end model for actual sequencing. We followed the guideline of extera XT DNA Sample Prep Kit, this kit use an enzymatic DNA fragmentation step, which allow to fragment and add adapter sequences onto template DNA with a single tube Nextera XT tagmentation reaction to generate multiplexed sequencing libraries. The tagmented DNA is amplified via a limited-cycle PCR program (12 cycles). MiSeq® Reagent Kit v3 (600 cycle) allows read lengths up to 2x300, this long read length is more likely to read over the junction (deletion for example) in most fragments (around 300bp) in one time cycle.

Mitochondrial DNA was loaded on the MiSeq (Illumina) for direct sequencing following standard protocols. The resulting data were analyzed using modified NextGENe software (SoftGenetics). This software was re-coded by scientists at SoftGenetics for our use in this project so that the software is better able to deal with mitochondrial DNA recombinant detection. The Revised Cambridge Reference Sequence of the Human Mitochondrial DNA was used as a reference. The scientists in SoftGenetics identified the fragments with gap by using modified NextGENe software, and then all individual positive fragments were further tested by using NCBI blast software, which revealed the exact gap information of each fragment.

### Detailed Analysis

We aligned the sequenced paired reads of each sample to the entire human genome with 93% similarity and separated the data into two parts: matched and unmatched reads. Mismatched reads are used for the rearrangement analysis. NextGENe software uses the human genome build 37 from NCBI including mtDNA as a reference. A 93% similarity in NextGene software gives a high confidence level because the software uses 12-mers to match to the reference and 6 mers to extend the alignment. The similarity is defined as matching score—mismatching score, with matching 1 point per base and mismatch penalty 2 points per base. This means that 93% is equivalent to 3.5% mismatching, or 7 mismatches for 200bp reads, when the mismatches are scattered in the reads. The coverage and consensus sequence for individual sample are calculated from the alignments of all reads to the mtDNA reference (Revised Cambridge Reference Sequence, NC_012920) with 85% similarity as a software default setting. The coverage of the samples at each base is reported in this step of the alignments. The coverage information of each base is comparable to a different sample using the gene bank reference. However, the consensus sequence of an individual patient was constructed to give the minimum number of SNP and to remove any systematic bias especially in the mtDNA hyper variable regions for future analysis. The origin of the circular genome was treated as two regions where a read is split into the two regions using supplementary alignment after software clipping the one end. Then all of the unmatched paired reads in the last step are aligned to the consensus sequence at 93% specificity. The matched paired reads are not used in the following analysis because these reads are from the individual mtDNA genome. When one end of the paired reads does not pass the 93% specificity, the paired reads are sorted into unmatched. The unmatched paired reads are further analyzed using NCBI blast.

GeneMarker HTS software has been used for SNPs analysis of mitochondrial DNA.Several specialized processing steps are used in order to improve alignment results: 3’ ends are soft-clipped when the base call quality is low. Illumina data is clipped when there are 3 consecutive bases with a quality less than Q25.This typically trims 35 to 40bp from each read in 300 bp PE Illumina libraries. The ends are soft-clipped when there are at least 2 mismatches in a 6 bp window, where multiple end mismatches are often due to residual of adapter/primer sequences, and novel structure change sequences. Potential alignment locations (including alignments across the origin) in the revised Cambridge Reference Sequence (NC_012920.1) are found using perfect matches to 27 bp hashes, and 12 bp extension and dynamic programming to fill the gaps. The final location is chosen based on the smallest number of mismatches. Both reads in a pair are aligned simultaneously. Alignments in specified ‘motif regions’ are adjusted to reflect traditional forensic variant calls that are based on phylogenetic analysis of known mitochondrial genomes in the world populations. Variant detection is based on simple (customizable) thresholds: 2% of reads contain the variant, the variant occurs in at least 10 reads, and there are at least 20 reads covering the variant position. Additional configurable filters use the median base call quality and directional allele balance to help to remove false positives. The difference in median base call quality between the two most common alleles in each direction at the position in the reads must be close, <10. The minimum directional allele balance ratio must be at least 25%—at least 25% of the reads containing the variant must be in the forward direction and at least 25% must be in the reverse direction.

Wilconxon of SAS was used to compare the rearrangement rate differences between ND and AD.

## Results

### Three types of mtDNA joining points have been classified

We detected thousands of mtDNA arrangement fragments from 13 AD patients and 12 aged matched controls (see S1 Data). Each rearrangement was defined by a unique combination of two joining points (5′ and 3′), the numbered of positions is according to the conventional light-strand (L-strand) of the revised Cambridge reference mtDNA sequence (rCRS; NC_012920). We classified these mtDNA rearrangements according to the difference of joining points as deletion, F-type rearrangement and R-type rearrangement. The 5′ joining points are upstream of the 5′ end, and 3′ joining points are downstream of the 3′ break using the L-strand numbering [8].

#### Mitochondrial DNA deletion (mtDNA deletion)

**Fig 1:**
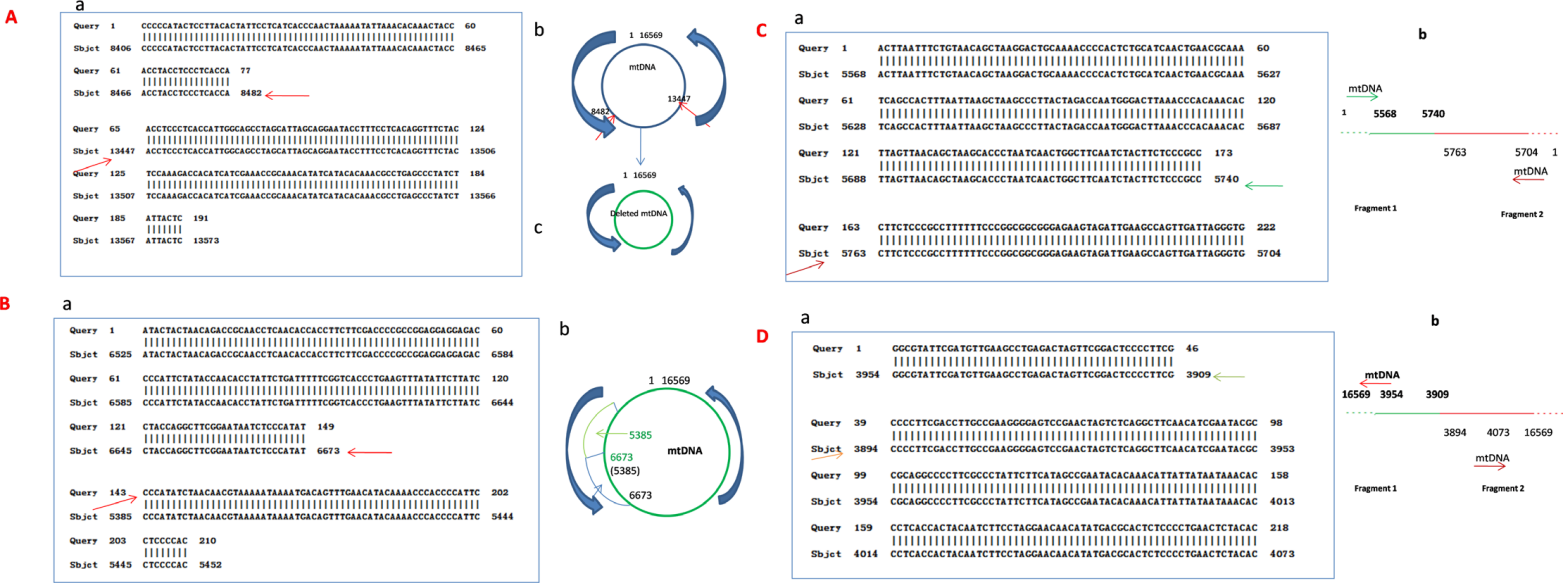
Example of mtDNA mtDNA recombinant. Blast result by using the sequence of fragments against the whole sequence of mtDNA genome. Query indicate the sequence of fragment, sbjct indicate the whole sequence of mtDNA genome. A. Deletion: Common mtDNA deletion 4977 between nt8482-13447 of mtDNA; a. indicate that a fragment between 8482 and 13447(red arrow) has been cut out, and a new small size mtDNA genome (deletion) has been formed as showed at b. B.F-type rearrangement: a.shows two fragments joining in the same direction, 5’ numbering is 6673, 3’ numbering is 5385 according to L-Strand. b. shows the model of F-type rearrangement, indicate the end of one fragment is 6673, and the end of another fragment is 5385, with the new mtDNA has duplicate sequence between nt5385-6673 (arrows show). C.R1-type rearrangement: a.shows two fragments jointed together to form new fragment with 5’ numbering is 5740, and 3’is 5763, the gap of numbering between 5’ joining point and 3’ joining point is 7,b.suggested model of R1 type rearrangement: one fragment from 1–5740 joined with another fragment in reverse orientation beginning is 5763 and end is 1. D. R2-type rearrangement: a.shows two fragments jointed together to form new fragment with 5’ numbering is 3909, and 3’ is 3894, the gap of numbering between 5’ breakpoint and 3’ breakpoint is 13, b.suggested the model of R2 type rearrangement: one fragment from 16569–3909 joined with another fragment, which is in reverse orientation beginning is 3894 and end is 16569.

**Fig 2:**
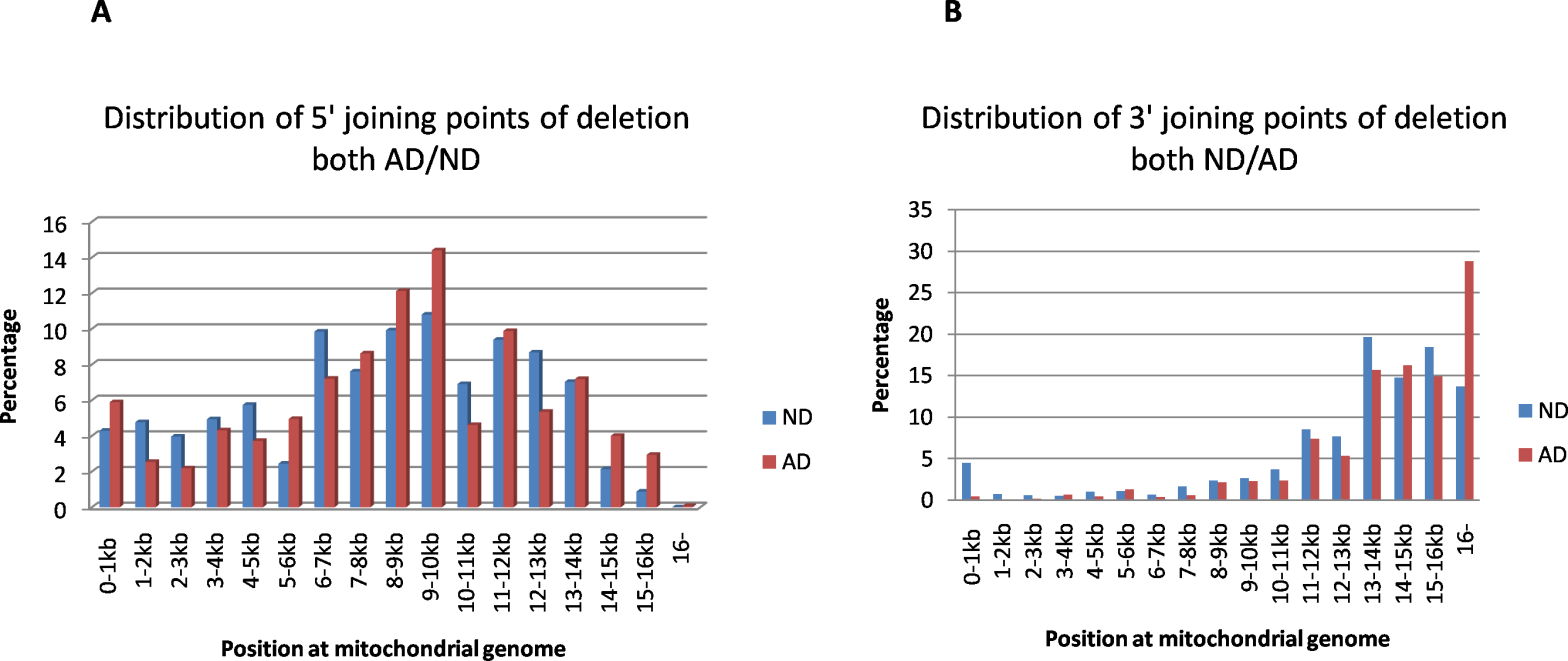
Distribution of 5’ and 3’joining points of deletion both ND and AD. A. 5’ joining points of deletion: The X-axis represents the nucleotide position along the mitochondrial genome divided into 1kb nucleotide increments, and the Y-axis represents the average percentage deletion part in aging samples (ND) or patients with Alzheimer’s disease (AD). The percentage of regional deletion within each kb is calculated by dividing the total number of deletions (5’ breakpoint) within 1kb by the total deletions in the sample. B. 3’ joining points of deletion: The X-axis represents the nucleotide position along the mitochondrial genome divided into 1kb nucleotide increments, and the Y-axis represents the average deletion part in aging samples (ND) or patients with Alzheimer’s disease (AD). The percentage of regional deletion within each kb is calculated by dividing the total number of deletions (3’ breakpoint) within 1kb by the total deletions in the sample. AD samples have more 3’ breakpoint at 16kb (28.8% in AD compared 13.7% in ND, P = 0.02).

The mtDNA deletion is defined by a combination of two reference numbers that identify the location of the 5’ and 3’ joining points, with the postulated recombinant lacking that part of the sequence of the mtDNA genome, see Fig 1: A mtDNA deletions accounted for 27% of the total detected rearrangement fragments in ND samples. The distribution of joining points of rearranged molecules is plotted in Fig 2. Both 5’ end and 3’ end joining points of deletions occur across the entire genome of mtDNA, with an uneven distribution. 5’ end joining points most frequently occur between 6-15kb of the mtDNA genome. The hotspot of 5’ deletion joining points is near 9kb-10kb, which is similar to literature reports^11^, and 3’ end joining points most frequently occur between 12kb and 16kb. AD samples have more 3’ joining points at 16kb and up (28.8% in AD compared to 13.7% in ND, P = 0.0326). Fig 2 show detail. The 5’ and 3’ joining points are often located within perfect or imperfect direct repeats; 79% of the identified deletions involved a direct repeat of at least 4 bp. Sizes of the deleted parts vary from a less than 5 to more than 1000 base pairs, with most being larger than 1kb. Small fragment deletions (<100bp) more frequently occur across positions 1-2kb of the mtDNA genome. The common mtDNA4977 deletion (nt8428-13447) was detected in most samples, but the percentage was quite low, only 1.5% of total detected deletion. Table 2 shows the frequency of some of the more common deletion types detected (those that appear in at least 5 of the 25 human subject samples). Again, the frequencies of these “common deletion” types are quite low.

**Table 2:** “Common deletions” detected from samples.

# of Deletion type	5’ joining point	3’ joining point	Frequency(percentage)	# of Deletion type	5’ joining point	3’ joining point	Frequency(percentage)
1	306	348	0.72	21	8629	14047	0.16
2	898	16285	0.5	22	8645	16565	0.14
3	1663	16141	0.16	23	8647	13373	0.08
4	2191	16108	0.36	24	8652	16073	0.14
5	3578	16184	0.48	25	9280	13064	0.12
6	3843	16100	0.36	26	9497	13723	0.46
7	4257	16211	0.18	27	9770	13449	0.12
8	5323	16109	0.36	28	11574	14079	0.1
9	6035	16106	0.12	29	11716	14936	0.26
10	6341	13989	0.34	30	11727	15430	0.34
11	6554	13827	0.22	31	12067	16158	0.2
12	7128	13992	0.12	32	12328	16243	0.14
13	7218	12109	0.18	33	12613	16133	0.2
14	7752	15822	0.14	34	13465	15979	0.14
15	7821	13573	0.1	35	13526	14947	0.26
16	8034	11423	0.38	36	13655	15263	0.18
17	8412	12380	0.24	37	13663	15406	0.48
18	8432	12084	0.14	38	14061	14360	0.1
19	8447	14056	0.2	39	14467	16151	0.18
20	8482	13447	1.51				

Note

Joining point at 5’ and 3’ are according to the conventional light-strand of the revised Cambridge reference mtDNA sequence (rCRS; NC_012920); Frequency (percentage): occurrence frequency of this type in the total deletions.

#### F-type rearrangement (tandem duplication)

**Fig 3:**
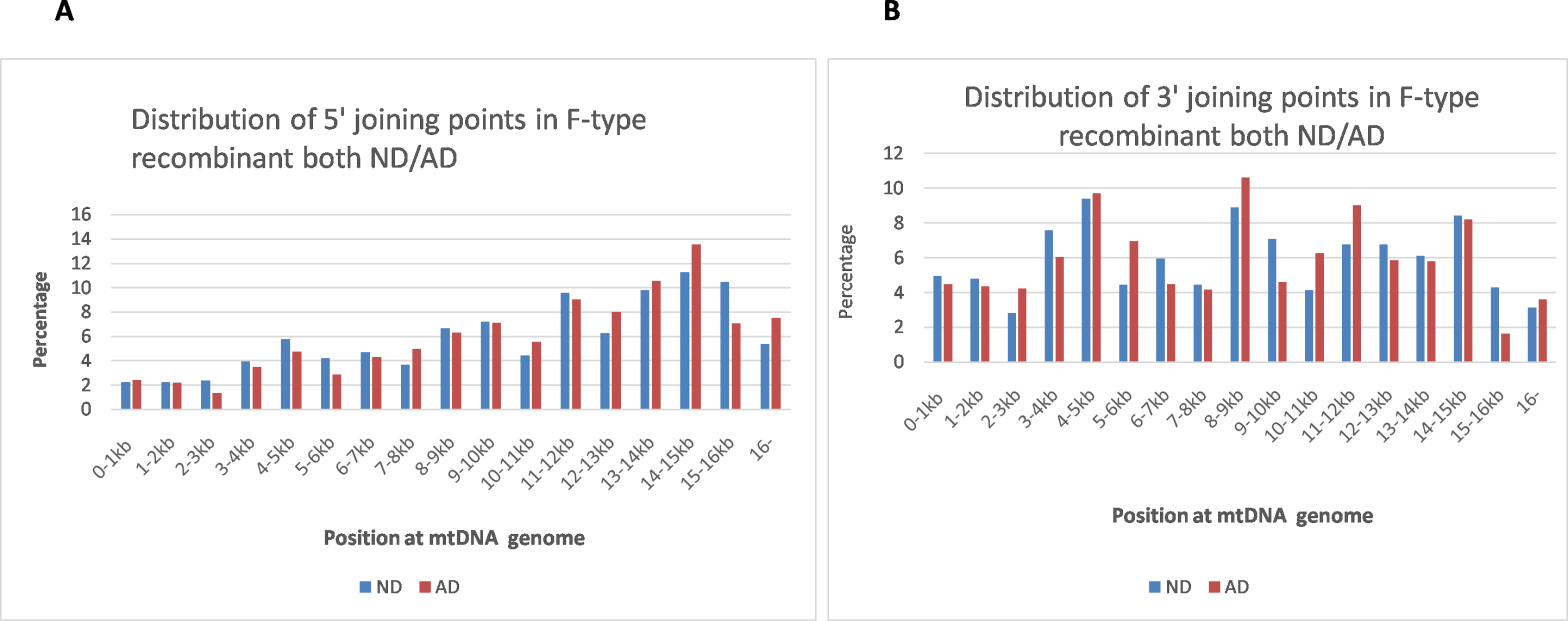
Distribution of 5’and 3’joining points in F-type rearrangement both ND and AD. A. 5’ joining points in F-type rearrangement: The X-axis represents the nucleotide position along the mitochondrial genome divided into 1kb nucleotide increments, and the Y-axis represents the average F-type rearrangement in aging controls (ND), or patients with Alzheimer’s disease (AD). The percentage of regional rearrangement within each kb is calculated by dividing the average total number of F-type breakpoint fragments within 1kb by the total average F-type fragments in the sample. B. 3’ joining points in F-type rearrangement: The X-axis represents the nucleotide position along the mitochondrial genome divided into 1kb nucleotide increments, and the Y-axis represents the average F-type rearrangement in aging controls (ND), or patients with Alzheimer’s disease (AD). The percentage of regional rearrangement within each kb is calculated by dividing the average total number of 3’ joining point fragments within 1kb by the total average F-type fragments in the sample.

F-type rearrangement is defined as fragments with two different sections of mtDNA joined together in the same direction; the postulated recombinant has a tandem duplicated sequence in some regions, which accounts for 19% of the total detected rearrangement fragments (see Fig 1B). Because the two joining fragments have the same orientation, we suggest this type of rearrangement might originate from duplicating partial mtDNA [9]. Fig 3 show the distribution characteristics of both the 5’ and 3’ end of joining points in F-type rearrangements, with uneven distribution across the whole mtDNA genome; both AD and control samples have similar distribution characteristics. Most of 3’ joining points (numbering according to L-strand positions of the revised Cambridge reference) in each rearrangement fragment are located upstream of the 5’ joining fragment, see Fig 1BB for example. The 5’ and 3’ joining points are often located within perfect or imperfect direct repeats; 82% of the identified deletions involved a direct repeat of at least 4 bp, See S1 Data for detail.

#### R-type rearrangement

**Fig 4:**
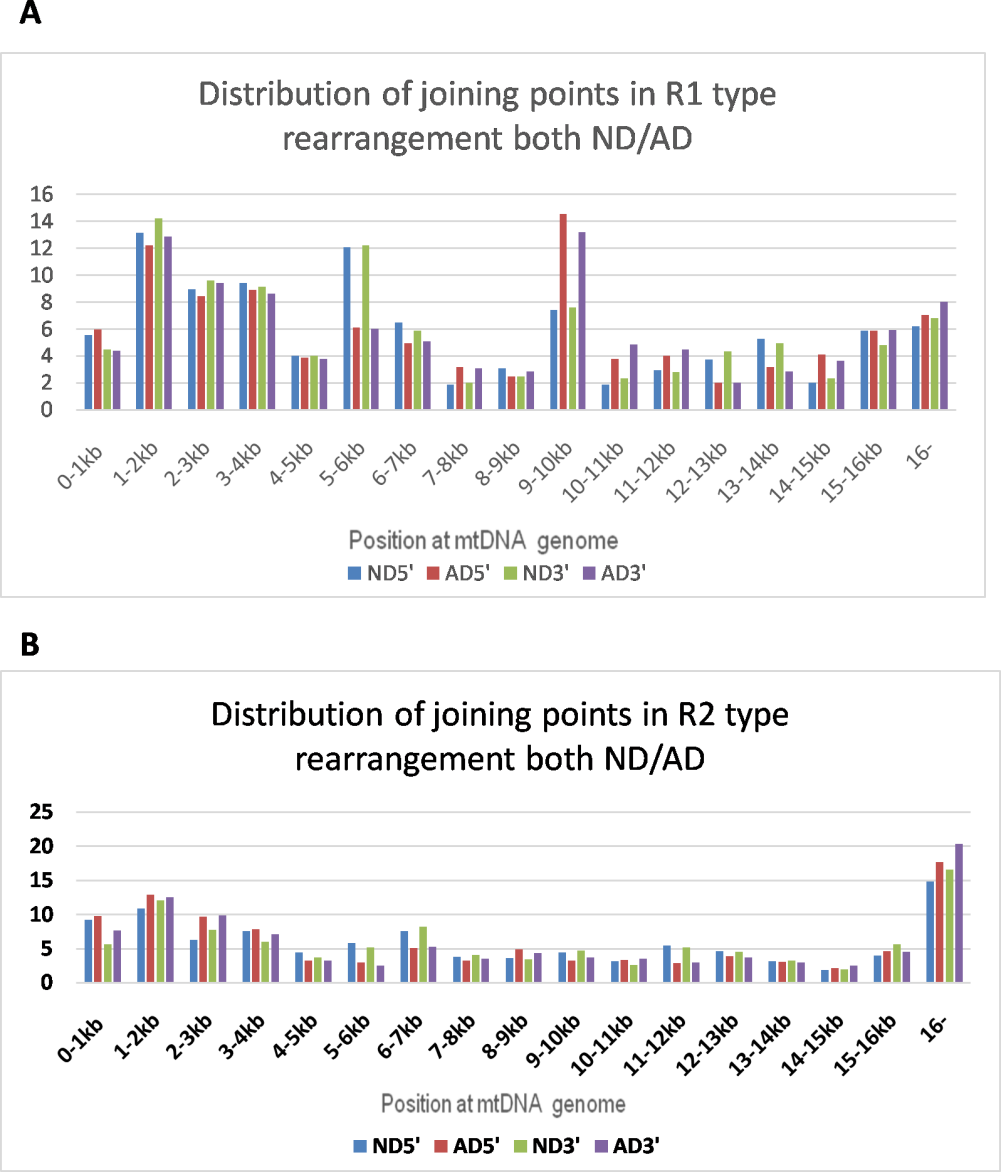
Distribution of both 5’ and 3’ joiningpointin R1-type or R2-type rearrangement. A. R1-type rearrangement: The X-axis represents the nucleotide position along the mitochondrial genome divided into 1kb nucleotide increments, and the Y-axis represents the average percentage of R1-type rearrangement in aging controls (ND), or patients with Alzheimer’s disease (AD). The percentage of regional rearrangement within each kb is calculated by dividing the average total number of 3’ or 5’ breakpoint fragments by the total R1-type fragments within 1kb in the sample. ND 5’ = 5’ breakpoint at ND; AD5’ = 5’ breakpoint at AD; ND 3’ = 3’ breakpoint at ND; AD 3’ = 3’ breakpoint at AD. There are more 5’ and 3’ joining points distributed at position 5-6kb(ND = 12.1%) compared with AD (AD = 6%), but p>0.5; there are there are more 5’ and 3’ joining points distribute at position 9-10kb for ND(7.5%) compared with AD(13.9%), but p>0.5. B. R2-type rearrangement: The X-axis represents the nucleotide position along the mitochondrial genome divided into 1kb nucleotide increments, and the Y-axis represents the average percentage of R2-type rearrangement in aging controls (ND), or patients with Alzheimer’s disease (AD). The percentage of regional rearrangement within each kb is calculated by dividing the average total number of 3’ or 5’ breakpoint fragments within 1kb by the total R2-type fragments in the sample. ND 5’ = 5’ breakpoint at ND; AD5’ = 5’ joining point at AD; ND 3’ = 3’ joining point at ND; AD 3’ = 3’ joining point at AD.

R-type rearrangement is defined as rearrangement of mtDNA originating from two different orientations of mtDNA fragments (L-strand or R-strand), which account for 54% of the total detected rearrangement fragments. There are two subtypes of R-type rearrangement: 1) R1-type, where the first joining fragment’s orientation is the same as the orientation of L-strand positions of the revised Cambridge reference mtDNA, with the second fragment joining in a reverse direction (Fig 1CC). Fig 4A shows the distribution of both 5’ and 3’joining points, which is uneven across the entire mtDNA genome, with two peaks, one is around 9-10kb, and another one is at the both ends of circle of mtDNA. 2) R2-type, where the first joining fragment’s orientation is the same as the orientation of H-strand positions of the revised Cambridge reference, with the second fragment joining in a reverse direction, see Fig 1DD) for example. Fig 4B shows the distribution of the 5’ and 3’ joining points, with most joining sites occurring at numbering 1 or 16569 of the mtDNA circle.

In R-type rearrangement, it seems that two close-vicinity fragments with different mtDNA orientation join together- one from the L-strand and another from the R-strand. The number of 5’ joining points and 3’ joining points within an mtDNA rearrangement are very close but with reversed orientation. The gap between 5’ and 3’ in most R-type rearrangements is less than 100bp, Fig 1C. Two common R-type recombinants have been detected, appearing in most samples: one belongs to an R1 type recombinant, its 5’ joining point beginning at 5763, while the 3’ reverse joining point begins at 5740 (this region is regarded as L-strand replication origin positions), see Fig 1C for detail. Another type is classified as R2 type recombinant, its 5’ joining points at 3909, and the 3’ reverse joining point begins at 3894 (See Fig 1D). Other data see S1 Data.

### Aging brain cells are rich in mtDNA rearrangements and AD brain tissue has markedly increased numbers

There were a total of 4046 fragments with mtDNA rearrangements detected in 12 individual control samples (mean age 82 yrs), with an average sequence coverage depth of 4262. There were a total of 8679 fragments with mtDNA rearrangements detected in 13 individual AD samples (mean age 80 yrs.), with an average sequence coverage depth of 2720. The positive number of rearrangement fragments and types of rearrangement increase with the coverage depth. Few rearrangements of mtDNA within the same sample (or even different samples in controls) share the same breakpoint type. In AD samples, some of recombinants share the same breakpoint in same or different subjects, but this concurrence frequency is quite low.

It is very difficult to calculate the exact rearrangement rate in each detected sample. Here, we introduce N/C rate and sub-N/C rate to compare the difference of mtDNA rearrangement among samples. The sub-N/C rate for deletion and F-type rearrangement is defined by the number of detected rearrangement fragments (joining point) of one sub-type rearrangement event divided by the average sequence coverage depth in each sample. The sub-N/C rate for R-type rearrangement is defined by the sum of detected R1 and R2 joining points of each sample divided by 2, then divided by the sequence coverage depth of this sample. The N/C rate is defined by the sum of sub N/C rate of deletion, F-type rearrangement, and R-type rearrangement in individual sample.

In ND control samples, the average total N/C rate is 6.7%. The average sub-N/C rate for deletion is 2.3%, for F type is 1.9%, and for R-type rearrangement is 2.6%. In AD samples, the total N/C rate is 17.9%, which is 2.7 times greater than that for ND controls (p = 0.0052); the sub-N/C rate for deletion in AD is 9.2%, which is 4 times that seen in ND samples (p = 0.0005); for F-type sub-N/C the rate is 4.17, which is 2.4 times that seen in ND samples (p = 0.0809); for R-type sub–N/C, the rate is 4.4%, 1.7 times that of ND controls (P = 0.6294).

### Heteroplasmy SNP analysis

As required by editor, we also determined the heteroplasmy SNPs in all of the samples of Alzheimer (AD) patients and control (ND).There are 13 samples for AD and 12 samples for ND. We have found 5 heteroplasmy positions shared at least two samples in either Alzheimer patients or control samples as shown in Table 3.The G71del, and A189G are shown in both types of the samples with the similar heteroplasmy mutation percentage. The C64T has been detected at 6 AD samples with average frequency is 2.3%, and detected at 4 ND samples with frequency is 6.8%. T72C shows heteroplasmy allele in 8 AD samples at average of 9.5% and standard deviation 4.2%, where it showed a minimum response in only 1 control sample at 2.1%. C16148T is shown only in two control samples at 2.5% and standard deviation 0.4%, but not in the AD patients. We have not found, in our samples, the heteroplasmy mutations of T414G and T477C and T146C reported in a literature [10]. See S2 Data.

**Table 3:** Heteroplasmy mutations shared at least two samples either in Alzheimer (AD) patients or control (ND) samples.

SNPs	Samples	#of Samples detected	Average frequency of SNPs	St dev
C64T	AD	6	2.3%	0.3%
C64T	ND	4	6.8%	9.4%
G71del	AD	10	7.1%	3.7%
G71del	ND	10	6.9%	4.9%
T72C	AD	8	9.5%	4.2%
T72C	ND	1	2.1%	
A189G	AD	12	4.6%	2.2%
A189G	ND	10	4.5%	1.8%
C16148T	AD	0		
C16148T	ND	2	2.5%	0.5%

Note: # of samples detected = Total numbers of samples has been detected in this type of SNPs from AD(N = 13),or ND (N = 12)

## Discussion

Mitochondrial dysfunction may play a central role in the process of aging and age-related diseases [11,12] such as AD, but there is still a scarcity of data that directly links the pathology of aging and/or AD with the alteration of mitochondrial DNA [13]. Combining direct sequencing of mtDNA from frozen brain tissue along with new DNA sequencing analysis software, for the first time, we provide a comprehensive assessment of mtDNA rearrangement events in aging cells. Differing numbers and types of mtDNA rearrangement fragments were detected from each sample depending on the sequencing coverage depth. There are few rearrangement fragments that share same joining point, but each sample shares similar distribution characteristics of joining points in mtDNA rearrangement. In additional, the distribution of deletion joining points in each sample is similar to previous reports [14]. These features indicate our data are valid reflections of the rearrangement status in these pooled mitochondrial DNA.

Very few rearrangement fragments share the same breakpoint in individual (control or AD) pooled mitochondrial DNA samples. It is probable that the occurrence of mtDNA rearrangement events is a semi-random process that is relatively unique to each cell. The short half-lives of mitochondria require a high rate of mtDNA replication in order to continually generate mitochondria. Over the lifespan this creates a risk of a large accumulation of abnormal mtDNA recombination, especially in long-lived postmitotic cells. Our results here are consistent with this concept.

We classified these rearrangement fragments as three types: deletion, F-type rearrangement (duplicate) and R-type rearrangement. To our knowledge, this is the first time R-type rearrangements have been identified. While mtDNA deletion mutations have been widely studied, only 730 types of deletion mutations have been identified over the past thirty years [14,15]. Here we showed a huge number of novel deletion mutations that exist in aging human specimens. In fact, as long as there is sufficient depth sequencing of the mtDNA genome, there are likely to be thousands of different deletion types in any brain sampled. Previously the most common mtDNA deletion has been the mtDNA4977 deletion (8470–8482/13447–13459). This was detected in our samples, but its frequency was quite low (1.5%) when compared with the total number of deletion types in each sample. We detected some deletion types that occur repetitively amongst samples, although still with individually low frequencies. These data suggest that using “common” deletions as markers of the overall deletion frequency is inappropriate. We also detected a large number of F-type rearrangements (duplicate). Only 37 types of duplicate of mtDNA have been detected in the past thirty years [14,15], but our data showed that this type of rearrangement is very common, with an uneven distribution across the whole mtDNA genome. R-type rearrangement, a newly identified type of mtDNA arrangement, is classified into two subtypes: R1-type rearrangement and R2-type rearrangement. The structure of R-type recombinants is unclear. There are two possible recombinants relative to R-type rearrangement: two different strands with different orientations but with complementary sequences (one from the L-strand, another from the H-strand) join together to form a linear recombinant with either an R1 or R2 breakpoint, or two fragments with different orientation may join together to form a circular recombinant (with both R1 and R2 joining points). Of these two possibilities the circular recombinant is more likely since mtDNA typically takes a circular form, but this needs further investigation. For this reason, we calculated the sub-R/C rate for R-type recombinants using a ratified sub-R/C: the sum of detected joining points of R1 and R2 divided by 2 as the number of detected joining points in R-type recombinants, which may more accurately reflect the recombinant status of mtDNA in one sample. The causes of rearrangement events are still unclear. It has been proposed that strand slippage during replication of mtDNA molecules leads to deletions in mtDNA [16], however a new hypothesis suggested that mtDNA deletions are created during the repair of double-stranded mtDNA [17]. For R-type mtDNA rearrangement, in which two differently oriented mtDNA strands join together, clarification must be sought as to whether replication error is the cause. Most deletions and duplications of mtDNA might be active and transcribable [18,19]. We do not know if R-type recombinants are transcribable. However, non-coding mitochondrial RNAs with long double-stranded, stem-loop structures have been detected within cells. It is possible that R-type mtDNA rearrangements are the origin of these double stranded mitochondrial RNAs [20,21].

It is difficult to accurately estimate the frequency of recombination of mtDNA, due to variability in fragment size and percentage of harboring cells. We used the total number of positive detected rearrangement fragments divided by the average sequence coverage depth in each sample (N/C rate) to assess the rearrangement level of that sample. The N/C rate cannot provide exact rearrangement levels, but should partially reflect the recombinant DNA status of each sample. In our control aging brain sample (average age 82, n = 12), the average N/C rate is 6.7%, which for first time provide evidence to support the “tip of the iceberg’ hypothesis, which assumes that mitochondrial DNA rearrangements are much more frequent than indicated by the frequency of common deletions such as the 4977 type [22]. Additionally, the observed high frequency of mtDNA rearrangements in brain tissue from aging donors may contribute to declining mitochondrial function with aging [23,24].

Evidence indicates that mitochondrial dysfunction has an early and preponderant role in Alzheimer's disease [25]. Our data supports this, as the AD brain samples had more than 2.7 times the recombinant rate of similarly-aged controls (P = 0.0052). Significantly, the sub-N/C rate for deletion in AD was 4 times that of the ND samples (p = 0.0005). We also found differences in the distribution of 3’ deletion joining points between AD and controls, with the AD samples having more 3’ joining points distributed found at 16kb or later. The high frequency of mtDNA rearrangements in AD allows that rearrangement events might be an upstream mechanism of the disease. Most recombinants (at least most of deletion and F-type rearrangement) might be transcriptionally active [18,19], but it is likely that their presence will result in the loss of some transcripts due to deletion while other transcripts might be over produced because of sequence duplication. The position of deletion joining points was not evenly distributed across the entire genome and instead was concentrated between regions 6kb and 15kb (Fig 2) of the mitochondrial genome, which happens to be the area containing the DNA sequences for synthesizing all three cytochrome oxidases. This makes it is reasonable to advance the concept that increased deletions in this area may affect the ability of mtDNA to synthesize cytochrome oxidase. Our results are consistent with reports of decreased cytochrome oxidase activity in AD brain samples [26,27]. Both strands of mtDNA are normally available for transcription. Heavy-strand transcription is initiated from two promoter sites, HSP1 and HSP2. It is thought that the initiation rate at the HSP1 site is 20-fold higher than at HSP2, which results in more transcripts of 12sRNA and 16sRNA in mitochondria [28,29]. Most deletion and F-type recombinants have intact sequences for transcribing 12sRNA and 16sRNA. It is reasonable to speculate that over-expression of ribosomal RNA may be present in aging and AD brain because of increased numbers of recombinants. It has been reported that mitochondrial 12S and 16S ribosomal RNA (rRNA) can fold chemically-denatured proteins and reactivate heat-induced aggregated proteins in vitro, which suggests that mitochondrial 12S and 16S ribosomal RNA play an important role in protein folding in the mitochondria [30,31], it is possible these abnormal accumulation of rRNA may interfere with protein folding in mitochondria since the vast majority of mitochondrial proteins are nuclear-encoded and must be imported into the mitochondria from the cytosol, which requires folding. Also, deficiencies of pyruvate dehydrogenase complex and ketoglutarate dehydrogenase [32] observed in AD brain may be caused by abnormal folding due to possible accumulation of ribosomal RNA. A comprehensive analysis of the human mitochondrial transcriptome shows that there is a rather large number of small transcribed mtRNA [33], which may play an important role in maintaining the synthesis of mtRNA and proteins. Old mitochondria must be destroyed through an autophagocytic pathway within days, which provides an excellent means for excess aberrant mtRNA to escape into the cytoplasm or nucleus [34]. Some non-coding mitochondrial RNAs with long double-stranded structures have been detected in the mitochondria or nucleus of cells, which might play important role in neoplastic transformation and cancer progression [20,21]. In addition, both L-strand and H-strand mtDNA have the ability to transcribe RNA, provide an opportunity for creating double-stranded mitochondrial RNA. Double-stranded RNA would be difficult to degrade by ribonuclease within the lysosome through the mitophagy pathway; however, there is a possibility that it may be processed by dicers or other similarendo-ribonucleases within the cytoplasm, and become a part of the iRNA system in cells [35]; participating in the regulation of gene expression at the transcription level.

## Supporting Information

S1 Data Rearrangements detected from samples. (XLSX)

S2 Data Heteroplasmy Spetrum in the control samples (ND) and Patients with Alzheimer’s disease (AD). (DOCX)
