# Supplementary material for: Mitochondrial DNA Rearrangement Spectrum in Brain Tissue of Alzheimer’s Disease: Analysis of 13 Cases
Source: PLoS One. 2016 Jun 14;11(6):e0154582. doi: 10.1371/journal.pone.0154582 (PMC4907522; doi:10.1371/journal.pone.0154582)
Supplement: S2 Data — (DOCX) [file pone.0154582.s002.docx]

Supplement Data2

Heteroplasmy Spetrum in the control samples (ND) and Patients with Alzheimer’s disease (AD).

Sample, Average Coverage, Heteroplasmy SNPs

ND01_31 18247 T72C(2.1%) A189G(3.4%) G7382A(3.3%) T16519C(2.1%) 298-348del

ND02_27 8617 G71del(4.9%) A189G(4.8%) G16213A(39%) 140-381del

ND03_47 1780 C64T(2.1%) A189G(5.5%) 298-348del

ND03_63 1743 G71del(2.3%) A189G(6.0%) C16148T(2.1%) 298-348del

ND06_21 1579 G71del(2.3%) A189G(3.1%) C524.1A(6.8%) C524.1C(6.8%) T16124C(68.3%) 298-348del

ND08_17 5485 G71del(9.6%) T119T(25.0%) G203A(6.0%) C309.1C(21.7%) T16243C(31.1%)

ND08_40 13123 G71del(3.5%) T9891C(4.7%) T16093C(49%)

ND09_57 3151 C64T(2.1%) G71del(5.7%) A189G(4.9%) C198T(7.3%)

ND10_20 7337 G71del(5.9%) A189G(3.4%)

ND10_39 1657 G71del(3.9%) A189G(8.5%) A8537G(34.4%) C10894T(62.3%) A16041G(59.4%) G16129A(71.2%) C16148T(2.9%) 298-348del

ND99_44 5420 T63T(19.9%) C64T(20.9%) G71del(18.6%) A189G(2.54%)

ND99_58 8056 C64T(2.26%) G71del(14.3%) A189G(2.27%) C16184.1C(24.7%)298-348del

AD00_37 1276 G71del(4.3%) A189G(5.7%) T5359C(8.1%)

AD01_53 2124 G71del(9.3%) A189G(5.2%) A10398G(88.1%) A14233G(9.4%)

AD02_28 7031 C64T(2.34%) G71del(8.4%) T72C(12.21%) A189G(3.4%) 298_348del

AD03_06 6751 C64T(2.07%) G71del(9.7%) A189G(6.1%) 298_348del

AD03_07 1009 A189G(2.4%) 298_348del

AD03_21 2391 C64T(2.5%) G71del(6.4%) T72C(11.6%) A189G(2.8%) T195C(48.8%) A5615G(4.9%) A14981T(7.1%) 298_348del

AD03_30 1389 T72C(2.4%) G3438C(2.7%) 298_348del

AD05_09 9028 T72C(3.7%) A189G(2.2%) C309.1C(9.3%) 298_348del

AD05_11 6444 C64T(2.1%) G71del(4.6%) T72C(10.5%) A189G(2.6%) 298_348del

AD10_16 1145 G71del(2.7%) A189G(4.5%) C16192C(17.3%) C16193.1C(15.7%)

AD11_12 4486 G71del(2.3%) A189G(4%) A16180d(3.0%)

AD11_27 5410 C64T(2.76%) G71del(11.9%) A189G(7.7%)

AD11_78 6618 C64T(2.14%) G71del(6.4%) T72C(10.8%) A189G(3.1%) 298_348del
